# Supplementary material for: Learning curve and surgical outcome of robotic assisted colorectal surgery with ERAS program
Source: Sci Rep. 2022 Nov 29;12:20566. doi: 10.1038/s41598-022-24665-w (PMC9709162; doi:10.1038/s41598-022-24665-w)
Supplement: Supplementary file 1 — Supplementary Information 1. [file 41598_2022_24665_MOESM1_ESM.zip › LSC Raw data-Ñ[▒K/114/N_ñw╝╨░O▒Kñσ.pdf]

W52 / W52

臺中榮民總醫院
手術室護理記錄

[redacted]

基本資料

- 室別： 二醫手術室-31室，手術科別： CRS，手術類別： 常規，日期： 2021/01/21
- 進入時間： 1545 ； 麻醉開始時間： 1545 ； Time Out時間： 1720 ； 劃刀時間： 1722 ； 手術結束時間： 2015 送出時間： 2020 ；
- 麻醉方式： GE
  診斷： Malignant tumor of sigmoid colon, 7CM IN SIZ, SP ATH, TRY LAP
  手術名稱： Laparoscopic Anterior resection and anastomosis - malignant
  手術局部用藥： nil
  術前預防性抗生素：
  - 抗生素名稱一： Veterin 1GM IVD由麻醉護理師給予
  - 時間： 1650
  - 上次簽章: 胡貞伶

  ，追加預防性抗生素：
- 手術醫師： 蔣鋒帆，林陳彥，陳映菁，江世偉，陳凱祥，黃瑋
- 麻醉醫師： VS 施涵薰
-
  - 刷手護士A ： 林宜旻，時間： 1545，接替刷手一： 張仟品，時間： 1730
  - 流動護士A ： 胡貞伶，時間： 1545，接替流動一： 林宜旻，時間： 1800

皮膚狀況

A-V Shunt： 無
術前： 完整
記錄者： 上次簽章: 胡貞伶
術後： 完整
記錄者： 林宜旻 上次簽章: 胡貞伶

手術準備

手術臥式 單一手術臥式

-
  - 手術臥式： 截石臥式
  - 使用附件： 溫毯 38 ℃，頭圈，腳架，其它： 頭架
  - 皮膚準備： 10%Aq Betadine，Hibiscrub，2% Chlorhexidine
  - 電燒： 使用電燒: 電極片位置： 其它： 迴路電極板，記錄者： 上次簽章: 胡貞伶

止血帶T： 無

植入物

- 植入物名稱/號碼/數量/部位： Surgiwrap 0.02x200x130mm/壹/骨盆腔

，記錄者： 林宜旻

標本送檢

- 細菌培養 無
- 細胞學 無
- 冰凍切片 無
- 病理標本
  組織名稱一： S-colon
  ，
  組織名稱二： Distal cutend
  ，
  組織名稱三： IMA root
  ，更多組織名稱
  - 組織名稱四： Para aortic LN
- 截肢 無

標本交付 標本： 肆 袋 ，標本： 零 盒 ，標本： 零 瓶 ，細胞學： 零 管 ，Culture Tube： 零 管 ，被交付醫師： R 呂曉瑜 ，

- 記錄者： 林宜旻
- 時間： 2020

傷口術後敷料

傷口敷料： 傷口敷料： 無

- 請於圖上標示部位：

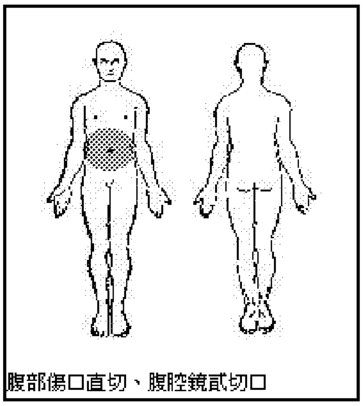

- 傷口敷料： 白紗

導管

導管	天數	導管資訊	插入日期	插入時間	更換日期	拔除日期	拔除時間	拔除原因	預計拔除/更換	預計拔除/更換日期
Penrose Drain(1)	1	號碼:12 mm.; 部位:骨盆腔;	2021/01/21	1900						
Sump NG Tube(1)	1	號碼:16 Fr.; 深度:55; 部位:胃;	2021/01/21	1700						
A-line (1)	1	號碼:22 ; 部位:前臂; 象限:LU;	2021/01/21	1630						
Foley(1)	1	號碼:16 Fr.; 部位:膀胱;	2021/01/21	1630						

用物計數

- 手術中出血量： minimal c.c.
- 手術中尿量： 650 c.c.
-
    - 其它： 無

用物計數： 多切口同時縫合

- 1.器械： 正確 ，2.縫針： 正確 ，3.刀片： 正確 ，4.紗布： 正確 ，5.腹部墊： 未使用 ，6.紗球： 未使用 ，7.PEANUTS ： 未使用 ，8.紗條： 未使用 ，9.絲帶： 未使用 ，10.針頭： 未使用 ，11.Vessel loop： 未使用 ，12.Boots： 未使用 ，13.Jelco： 未使用 ，14.棉條： 未使用 ，
    - 第一次：
        - 刷手護士 ： 張仟品
        - 流動護士： 林宜旻
        - 時間： 1925

  ,
    - 第二次：
        - 刷手護士 ： 張仟品
        - 流動護士： 林宜旻
        - 時間： 1940

  ,
    - 第三次：
        - 刷手護士 ： 張仟品
        - 流動護士： 林宜旻
        - 時間： 1950

備註

備註： NIL

- 記錄者： 林宜旻 上次簽章: 林宜旻
- 時間： 2015

填寫護理人員 林宜旻

正式摘要

W52 / W52

doctorRank:B
